# Supplementary figures and images for: Polycyclic Aromatic Hydrocarbon Risk Assessment and Analytical Methods Using QuEchERS Pretreatment for the Evaluation of Herbal Medicine Ingredients in Korea
Source: Foods. 2021 Sep 16;10(9):2200. doi: 10.3390/foods10092200 (PMC8472304; doi:10.3390/foods10092200)

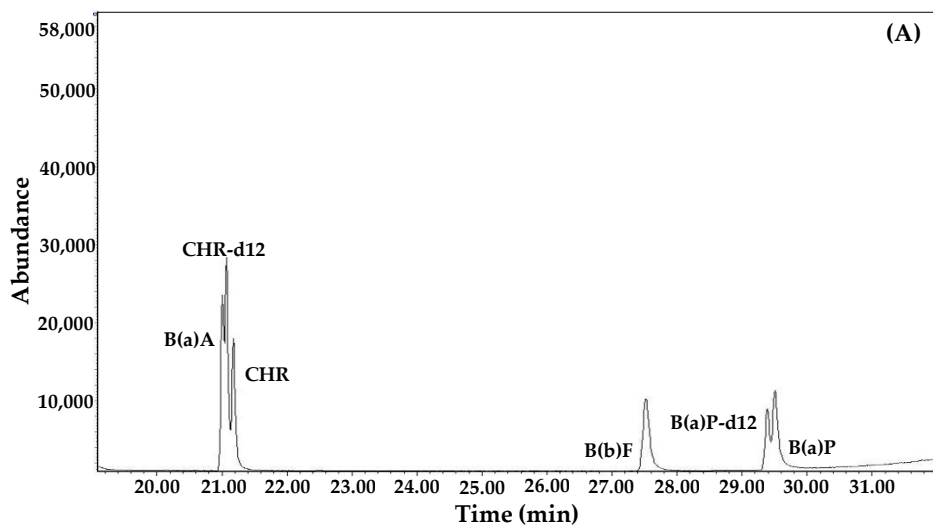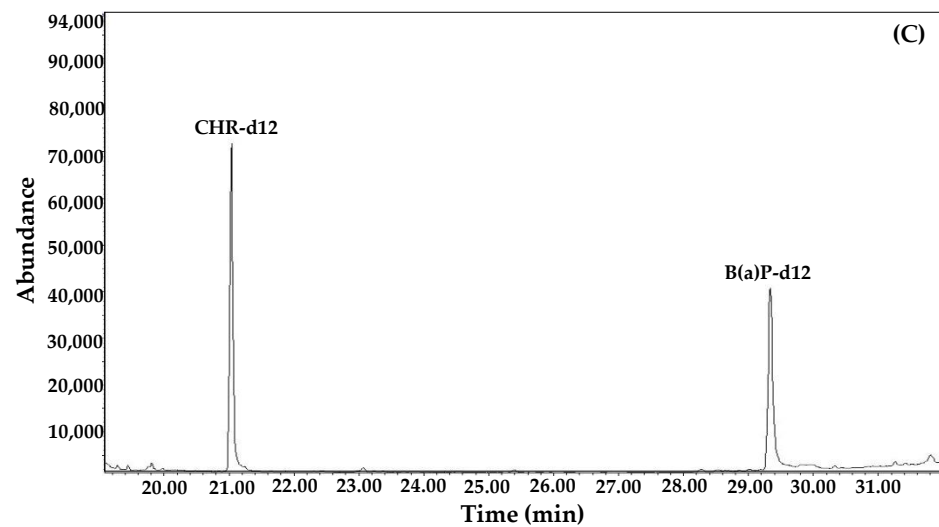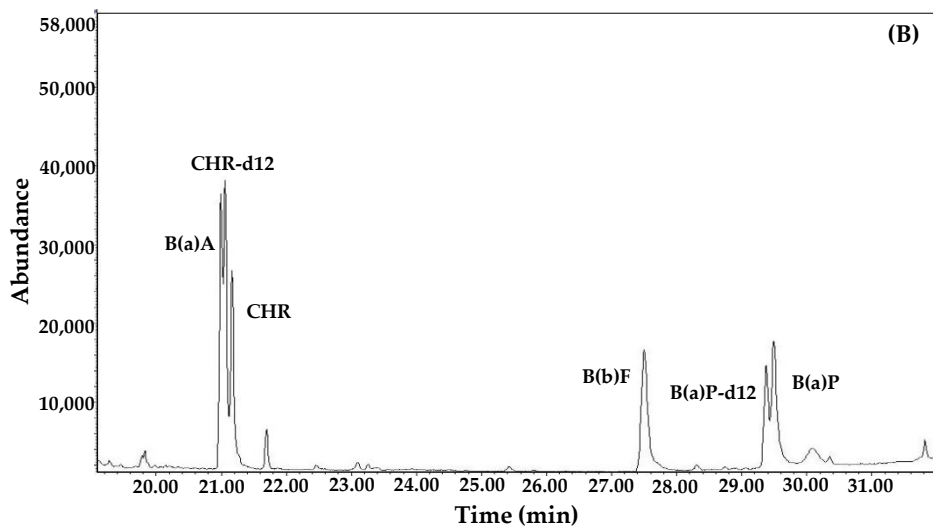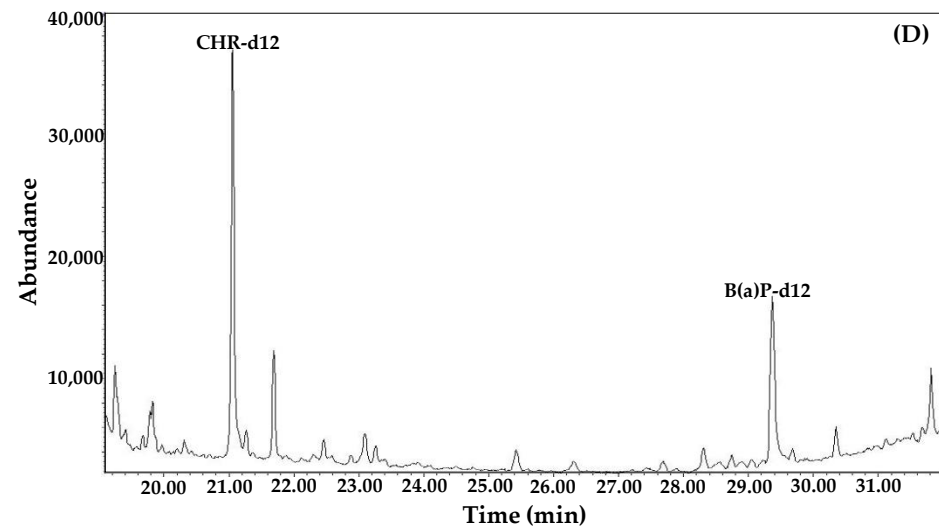

Supplement: Supplementary file 1 [file foods-10-02200-s001.zip › foods-1365613- Supplementary figures/Figure S1.pdf]

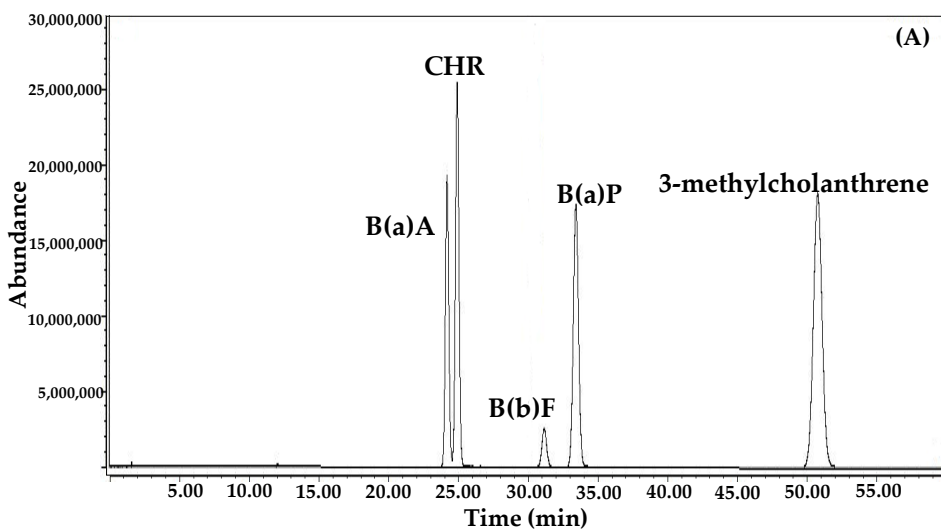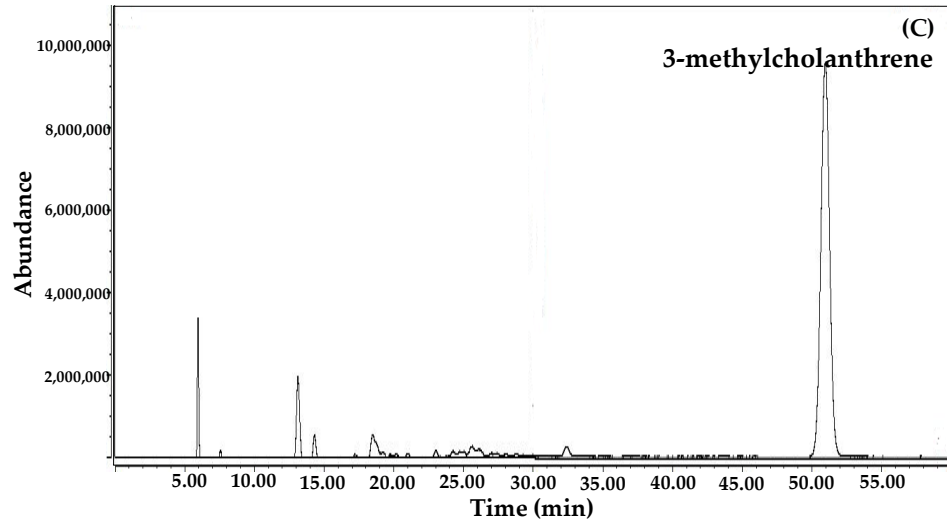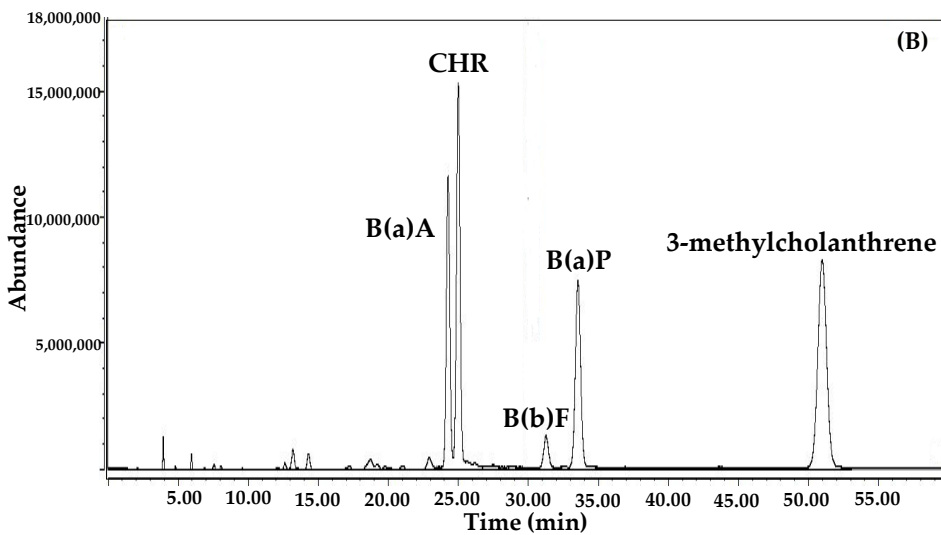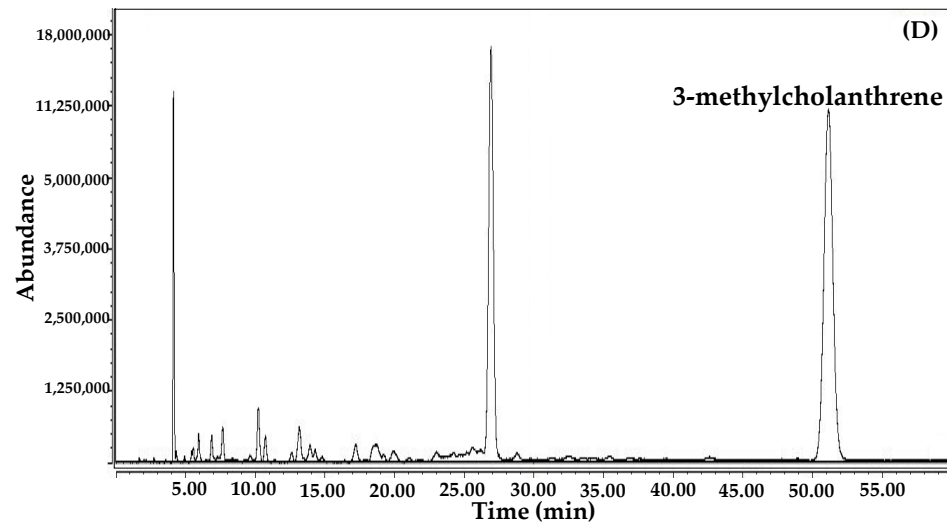

Supplement: Supplementary file 1 [file foods-10-02200-s001.zip › foods-1365613- Supplementary figures/Figure S2.pdf]
